# Physico-chemical variables determining the invasion risk of freshwater habitats by alien mollusks and crustaceans

**DOI:** 10.1002/ece3.382

**Published:** 2012-10-11

**Authors:** Denise Früh, Stefan Stoll, Peter Haase

**Affiliations:** Department of River Ecology and Conservation, Biodiversity and Climate Research Centre & Senckenberg Research Institute and Natural History Museum FrankfurtClamecystr. 12, 63571, Gelnhausen, Germany

**Keywords:** Alien species, benthic invertebrates, distance, physico-chemical degradation, temperature

## Abstract

The aim of this study was to assess the invasion risk of freshwater habitats and determine the environmental variables that are most favorable for the establishment of alien amphipods, isopods, gastropods, and bivalves. A total of 981 sites located in streams and rivers in Germany. Therefore we analyzed presence–absence data of alien and indigenous amphipods, isopods, gastropods, and bivalves from 981 sites located in small to large rivers in Germany with regard to eight environmental variables: chloride, ammonium, nitrate, oxygen, orthophosphate, distance to the next navigable waterway, and maximum and minimum temperature. Degraded sites close to navigable waters were exposed to an increased invasion risk by all major groups of alien species. Moreover, invaded sites by all four groups of alien species were similar, whereas the sites where indigenous members of the four groups occurred were more variable. Increased temperature and chloride concentration as well as decreased oxygen concentration were identified as major factors for the invasibility of a site. Species-specific analyses showed that chloride was among the three most predictive environmental variables determining species assemblage in all four taxonomic groups. Also distance to the next navigable waterways was similarly important. Additionally, the minimum temperature was among the most important variables for amphipods, isopods, and bivalves. The bias in the occurrence patterns of alien species toward similarly degraded habitats suggests that the members of all four major groups of freshwater alien species are a non-random, more tolerant set of species. Their common tolerance to salinity, high temperature, and oxygen depletion may reflect that most alien species were spread in ballast water tanks, where strong selective pressures, particularly temperature fluctuations, oxygen depletion, and increased salinity may create a bottleneck for successful invasion. Knowledge on the major factors that influence the invasion risk of a habitat is needed to develop strategies to limit the spread of invasive species.

## Introduction

Biological invasions are a significant component of human-caused global change and one of the major threats to biodiversity of terrestrial, marine, and freshwater systems (Lövei 1997; Dextrase and Mandrak 2006). Beside the negative impact on biodiversity, invasions also accrue high economic costs (Pimentel et al. 2005). Especially, freshwater systems are affected by alien species (Sala et al. 2000), due to their high interconnectedness via man-made waterways and shipping (Gollasch and Nehring 2006). The extensive network of inland waterways in combination with increasing shipping intensities has led to a sharp increase in invasion during the last 50 years. Both the development of navigable inland waterways and the tonnage of transported goods are predicted to further increase (EC 2006), leading to the expectation that also the introduction rate of alien species will increase.

In order to predict and possibly inhibit future invasions, it is important to understand the factors that mediate invasion processes. Among these factors are the conditions of the new habitat, favorable traits of the alien species for successful establishment in the new habitat, and human-cause factors.

In this context, anthropogenic introduction is one of the most important control factors in biological invasion. In addition to the ornamental trade and stocking, transport via shipping and man-made waterways is the most important vector for alien species (Gollasch and Nehring 2006). Thus, streams and connected riverine systems, especially those with extensive navigation, act as corridors for the dispersal of alien species and are highly infected by alien species. For example, the River Rhine, the busiest waterway of the world, harbors more than 45 alien invertebrate species (Leuven et al. 2009).

The set of aquatic species that can benefit from these pre-dispositions to invasions is a non-random selection of all aquatic species. Especially, mollusks and crustaceans are over-represented (Karatayev et al. 2009; Strayer 2010). In this context, it has been shown that alien species often are characterized by life history traits that may facilitate successful establishment. For instance, Grabowski et al. (2007) showed for alien gammarids that they are characterized by a combination of large brood size, high partial fecundity, early maturation, and a high number of generations per year.

Beside favorable life history traits, it has been shown for a range of alien species that in comparison with indigenous species, aliens are often characterized by a higher tolerance to habitat degradation (Havel et al. 2005; Johnson et al. 2008), increased temperature (Wijnhoven et al. 2003; Werner and Rothhaupt 2008; Weitere et al. 2009; Zukowski and Walker 2009; Sargent et al. 2011), and chemical degradation, such as oxygen depletion (Byers 2000a; MacNeil et al. 2000) or an increase in salinity (Grabowski et al. 2009). These characteristics were mostly analyzed in laboratory or field studies on single species (e.g., Werner and Rothhaupt 2008; Weitere et al. 2009) or species groups, like gammarids (Grabowski et al. 2009). However, comparative studies investigating several alien taxa are rare. Nevertheless, Karatayev et al. (2009) showed in their study of a large set of alien species that they tolerate at least moderate loads of organic pollution. The increased tolerances of these alien species to degradation make degraded systems more vulnerable to invasion (Schreiber et al. 2003; MacDougall and Turkington 2005; Früh et al. 2012). However, there are two ways in which alien species manage to establish themselves, especially in degraded habitats. Alien species may outcompete indigenous species in degraded habitats, or more tolerant alien species may fill the resulting gap left by indigenous species after extermination from their habitats by various stressors (Didham et al. 2005; MacDougall and Turkington 2005).

Although the favorable life history traits and the vectors of alien species are well known, knowledge on the environmental variables that affect the invasibility of a habitat is still incomplete. Although it is known that urban (Pysek et al. 2010), nutrient-rich (Vermonden et al. 2010) and degraded habitats (Schreiber et al. 2003; MacDougall and Turkington 2005; Früh et al. 2012) are more easily invaded, the role of single environmental variables determining the invasibility of freshwater habitats is still poorly investigated. Moreover, it remains unclear if the same environmental variables are important for different groups of aquatic alien species. However, to improve the understanding and prediction of invasion patterns, it is important to identify general determinants of invasion risk of freshwater habitats. The assessment of invasion risk of habitats is a fundamental component in order to monitor current invasion as well as to develop actions to control and prevent future invasion.

Here, we analyzed an extensive dataset to assess the invasion risk of freshwater habitats by gastropods, bivalves, amphipods, and isopods, which comprise the highest number of alien invertebrates in freshwater systems (Strayer 2010). As in freshwater habitats, shipping is the main vectors for alien species' introduction (Gollasch and Nehring 2006), the propagule pressure, which plays the driving role in invasion processes (Lockwood et al. 2005, 2009), differs between navigable and non-navigable waters.

Due to the constant delivery of propagules in combination with a high degree of anthropogenic habitat alterations, most of the navigable waters are already invaded by one or several alien species. Invaded habitats are also more prone to additional invasions, which can lead to invasion meltdowns (Simberloff and Holle 1999). From navigable waters as sources, a spread of invasive species to the tributary systems is currently being observed. In this study, we analyze the risk of such tributary systems to become invaded.

Particularly, the following questions were asked:

1. Are degraded freshwater habitats more vulnerable to invasion? – Are there consistent patterns of environmental variables that determine the invasion risk of alien amphipods, isopods, bivalves, and gastropods?
2. Which variables are the most important predictors for invasion risk of freshwater habitats by the different groups of alien species?
3. Which variables determine the composition of species assemblage within the four different groups: amphipods, isopods, bivalves, and gastropods?

## Methods

### Benthic macroinvertebrate data

**Figure 1:**
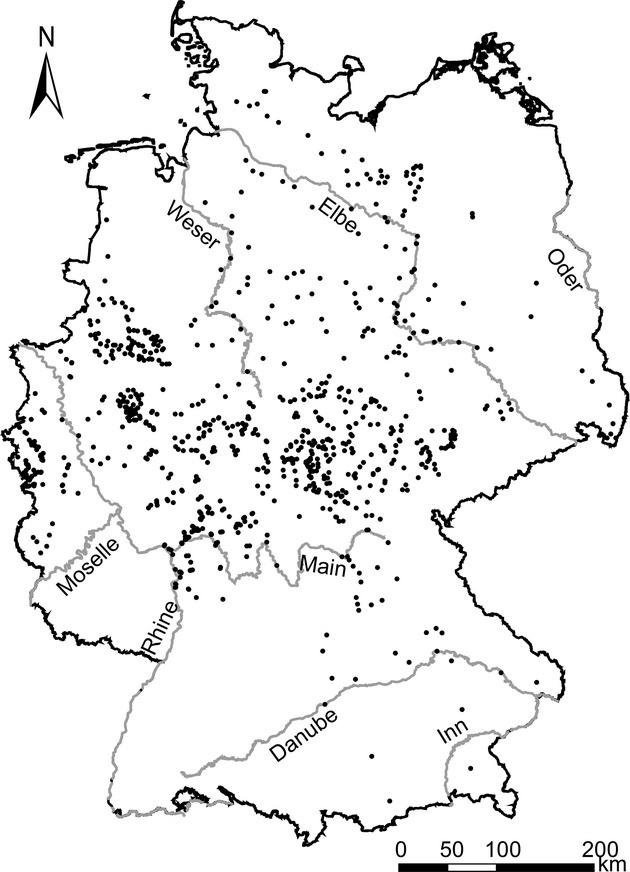
Map of the location of the 981 sampling sites located in small to large rivers in Germany. Additionally, the eight largest German rivers are indicated in the map.

The benthic invertebrate data were derived from several databases (unpublished data from Umweltbundesamt, Hessisches Landesamt für Umwelt und Geologie, EU-STAR project). Data from a total of 981 sampling sites located in small to large rivers in Germany with a catchment area from 10 to 10,000 km^2^ could be obtained (Fig. 1). Sixty-nine of the sites were located in navigable waters, 912 in non-navigable waters.

The data were collected between February and October from 2000 to 2008, following the official European Water Framework Directive compliant sampling protocol. A multi-habitat sampling approach was used: at each site, 20 sub samples were taken, covering all microhabitat types according to their relative occurrence. Each subsample was notionally of 25 cm × 25 cm in size, resulting in ca. 1.25 m^2^ of river bottom being sampled. The samples were preserved in 70% ethanol and transferred to the laboratory where they were sorted following the method of Haase et al. (2004). Taxa were identified to the level proposed by Haase et al. (2006) ensuring that taxalists were inter-comparable with regard to their taxonomic resolution.

The species were classified as indigenous species or alien species based on the species list published by (DAISIE 2011); the term alien species is used according to the definition by (Genovesi and Shine 2004).

### Environmental variables

For most of the benthic invertebrate sampling sites, direct measurements of Cl, NH_4_, NO_3_, O_2_, and PO_4_were available from several databases (unpublished data from Umweltbundesamt, Hessisches Landesamt für Umwelt und Geologie, EU-STAR project).

Where measurements of these chemical variables taken directly at the sampling sites were lacking, representative measuring stations for the sites were selected according to the following criteria: (a) the time when the measurements were taken had to correspond to the time of benthic invertebrate sampling; (b) the measuring station to be within a radius of 2 km around the invertebrate sampling site; and (c) no tributary or other water inlet was allowed, to be located between the measuring station and the sampling site.

In addition to the chemical variables, we used two temperature variables: the mean of the warmest quarter of the year (BIO10, hereafter referred to T_max_), and mean of the coldest quarter of the year (BIO11, hereafter referred to T_min_) from the Worldclim database with a spatial resolution of 30 arc sec (approx. 1 × 1 km) (Hijmans et al. 2005).

As shipping is an important vector for most alien species (Gollasch and Nehring 2006), the distance to the next navigable waterway may be used as a proxy for the colonization and propagule pressure of alien species to the sites. Therefore, for every sampling site, we calculated the distance to the next navigable waterway. This variable allows differentiation between physico-chemical driven patterns and colonization pressure, which decrease with increasing distance to the next navigable waterway. The calculations were performed in GIS desktop (ESRI Inc, Redmond, USA) using the spatial analyst tool “cost distance”.

### Statistical analysis

To analyze the invasion risk of the sampling sites, they were divided into two groups: sites where just indigenous species (648) were found, and sites where also alien species (333) occurred. To identify taxonomically unrelated patterns, these groups were further subdivided in sites where species of amphipods, isopods, gastropods, and bivalves were found, resulting in eight classifications of sampling sites.

Data for chemical variables (Cl, NH_4,_ NO_3,_ O_2_, and PO_4_) were log(x + 1)-transformed.

Invasion risk of sampling sites with regard to general environmental conditions was analyzed using the principle component (PC) scores determined by principle component analysis (PCA). After having checked, the length of gradient using detrended correspondence analysis (DCA), we condensed the multiple spaces of the environmental variables of all sampling sites using a PCA. Multivariate statistics were performed in PC-ORD 5 for Windows (MjM, Software Design, Gleneden Beach, Oregon, USA).

First, we compared the scores of all significant PCs (1–3) of invaded and non-invaded sites using Mann-Whitney *U*-tests (MW*U*-tests). As PC3 did not differ significantly between invaded and non-invaded sites, it was not considered in further analysis. Therefore, we used PC1 and PC2 scores to analyze the invasibility of the sites by all groups of alien species with regard to physico-chemical degradation and distance to the next navigable waterway.

We compared the means of the PC1 and PC2 scores for all eight groups of sites using Kruskal-Wallis ANOVA including multiple comparisons. PC scores were considered as dependent variables and the classification of sites as the grouping variable.

To identify the role of single environmental variables for invasibility, we used t-tests to calculate pairwise comparisons between environmental variables of the sites where alien species of the four groups (amphipods, isopods, gastropods, and bivalves) occurred and the sites where just indigenous species of the corresponding groups occurred. The measures of environmental variables were considered as dependent and the classification of the sites (alien species groups vs. indigenous species groups) as the grouping variable. To avoid α-error inflation, significant *P*-values were adjusted using the Bonferroni-Holm method. All univariate analyses were performed using STATISTICA 8 (StatSoft, Inc., Tulsa, USA).

To analyze the contributions of the single environmental variables to the composition of alien and indigenous species assemblages, we calculated canonical correspondence analysis (CCA) for each taxonomical group. Prior to this analysis, we checked the lengths of gradients using a DCA. As the length of gradients was > 3 for all taxonomic groups, we chose unimodular response models to relate the presence–absence data of the alien and indigenous species to the environmental variables. The significance of environmental variables for determining the species composition was tested using a forward selection and 999 Monte Carlo permutations under full model conditions. For these species-specific analyses, a subset of the dataset was used. Just species with at least five sampling sites were considered (Table 1).

**Table 1:** List of alien (AS) and indigenous (IS) species, including abbreviations, of the four different taxonomical groups, amphipoda (AMP), isopoda (ISO), gastropods (GAS), and bivalves (BIV), which were considered in the species-specific analysis. Just species with at least five sites of occurrence were considered

AS/IS	Group	Species	Abbreviations
AS	AMP	*Corophium curvispinum*	*C.cur*
AS	AMP	*Dikerogammarus villosus*	*D.vil*
AS	AMP	*Echinogammarus berilloni*	*E.ber*
AS	AMP	*Gammarus tigrinus*	*G.tig*
AS	BIV	*Corbicula fluminea*	*C.flu*
AS	BIV	*Dreissena polymorpha*	*D.pol*
AS	GAS	*Physella acuta*	*P.acu*
AS	GAS	*Physella heterostropha*	*P.het*
AS	GAS	*Potamopyrgus antipodarum*	*P.ant*
AS	ISO	*Jaera istri*	*J.ist*.
AS	ISO	*Proasellus coxalis*	*P.cox*
IS	AMP	*Gammarus fossarum*	*G.fos*
IS	AMP	*Gammarus pulex*	*G.pul*
IS	AMP	*Gammarus roeselii*	*G.roe*
IS	BIV	*Anodonta anatina*	*A.ana*
IS	BIV	*Musculium lacustre*	*M.lac*
IS	BIV	*Pisidium amnicum*	*P.amn*
IS	BIV	*Pisidium casertanum*	*P.cas*
IS	BIV	*Pisidium henslowanum*	*P.hen*
IS	BIV	*Pisidium milium*	*P.mil*
IS	BIV	*Pisidium nitidum*	*P.nit*
IS	BIV	*Pisidium personatum*	*P.per*
IS	BIV	*Pisidium subtruncatum*	*P.sub*
IS	BIV	*Pisidium supinum*	*P.sup*
IS	BIV	*Sphaerium corneum*	*S.cor*
IS	BIV	*Sphaerium rivicola*	*S.riv*
IS	BIV	*Unio pictorum*	*U.pic*
IS	BIV	*Unio tumidus*	*U.tum*
IS	GAS	*Acroloxus lacustris*	*A.lac*
IS	GAS	*Ancylus fluviatilis*	*A.flu*
IS	GAS	*Anisus vortex*	*A.vor*
IS	GAS	*Bathyomphalus contortus*	*B.con*
IS	GAS	*Bithynia tentaculata*	*B.ten*
IS	GAS	*Galba truncatula*	*G.tru*
IS	GAS	*Gyraulus albus*	*G.alb*
IS	GAS	*Lymnaea stagnalis*	*L.sta*
IS	GAS	*Physa fontinalis*	*P.fon*
IS	GAS	*Planorbarius corneus*	*P.cor*
IS	GAS	*Planorbis carinatus*	*P.car*
IS	GAS	*Planorbis planorbis*	*P.pla*
IS	GAS	*Radix auricularia*	*R.aur*
IS	GAS	*Radix balthica*	*R.bal*
IS	GAS	*Radix labiata*	*R.lab*
IS	GAS	*Theodoxus fluviatilis*	*T.flu*
IS	GAS	*Valvata cristata*	*V.cri*
IS	GAS	*Valvata piscinalis*	*V.pis*
IS	GAS	*Viviparus contectus*	*V.con*
IS	GAS	*Viviparus viviparus*	*V.viv*
IS	ISO	*Asellus aquaticus*	*A.aqu*

## Results

With about 90% (60 of 69), most sites in navigable waters were invaded by alien species, whereas in non-navigable waters, just about 30% of sites (273 of 912) were invaded. As in navigable waters, no substantial number of non-invaded sites was available, we limited the further analysis on variables affecting invasion risk to non-navigable waters.

### Are degraded freshwater habitats more vulnerable to invasion? – Are there consistent patterns of environmental variables that determine the invasion risk of alien amphipods, isopods, bivalves, and gastropods?

The comparison of PC scores showed that invaded and non-invaded sites differed with respect to their general physico-chemical degradation and distance to the next navigable waterway. PC1 to 3 were found to extract a significant proportion of the variability in the dataset (Table 2).

**Table 2:** Results of the principle component analysis (PCA) calculated for environmental variables of invaded (AS) and non-invaded sites (IS), randomization tests (999 runs) and Mann-Whitney *U*-test (MW*U*-test) of the principle component (PC) scores for AS versus IS sites. The eigenvalues, extracted variances (%), *P*-values, and correlation coefficients are given for the significant PCs. O_2_, PO_4_, Cl), NO_3_, NH_4_, mean of the warmest quarter (T_max_), mean of the coldest quarter (T_min_), distance to the next navigable waterway (Distance); n.s. = not significant

	PC1	PC2	PC3*
% of variance	30.72	19.41	13.97
Eigenvalues	2.46	1.55	1.12
*P*	0.001	0.001	0.001
Eigenvectors of Variable			
O_2_	0.54	0.15	0.53
Cl	−0.62	0.25	−0.02
PO_4_	−0.45	0.67	0.04
NO_3_	−0.20	0.41	0.77
NH_4_	−0.29	0.72	−0.36
T_max_	−0.76	−0.24	−0.05
T_min_	−0.62	−0.43	0.32
Distance	0.71	0.31	−0.10
AS versus IS (MW*U*-test)	*P* < 0.001	*P* = 0.02	n.s.

* As the scores of PC3 did not differ between AS and IS sites, these PC scores were not considered in further analysis.

**Figure 2:**
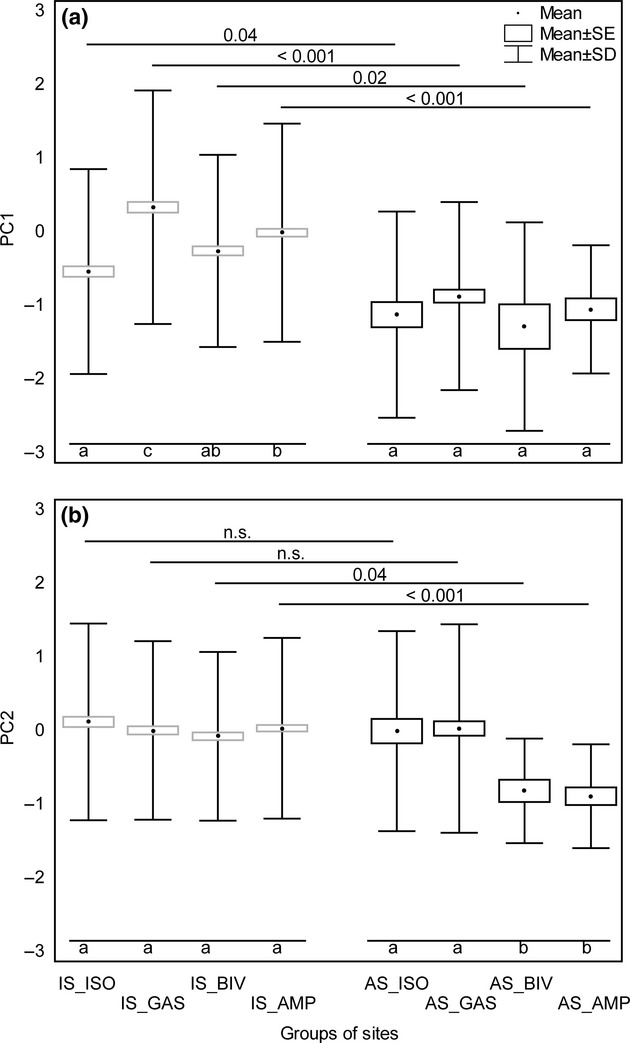
Box plots of the principle component (PC) scores calculated with principle component analysis (PCA) for PC1 (a) and PC2 (b). Mean ± SE and ± SD are given. The results comparing invaded (AS) and non-invaded (IS) sites for isopods (IS_ISO; AS_ISO), gastropods (IS_GAS; AS_GAS), bivalves (IS_BIV; AS_BIV) and amphipods (IS_AMP; AS_AMP) are shown. Letters indicate significant differences in Kruskal-Wallis ANOVA between the sites of the single groups of AS and IS, respectively. *P*-values indicate significant differences in pairwise comparisons between the sites of IS groups vs. the corresponding AS groups (n.s. = not significant).

The PC1 explained about 31% of the variability in the dataset and was inversely correlated with all variables indicating environmental degradation: Cl, PO_4_, NO_3_, NH_4_, T_max_, and T_min_ (Table 2). The highest negative correlation was found for T_max_, T_min_, and Cl. At the same time, PC1 was positively correlated to O_2_ concentration, an indicator for good habitat quality, as well as to distance to the next navigable waterway. Thus, this PC represented a double gradient of degradation as well as propagule pressure. Pristine sites and distant sites to navigable waters with low propagule pressure were located at high PC1 values, whereas degraded sites and sites close to navigable waters with high propagule pressure were located at low PC1 values. In all four taxonomic groups (amphipods, isopods, gastropods, bivalves), invaded sites compared with non-invaded sites were oriented toward higher degradation and closer distance to the next navigable waterway. Furthermore, the PC1 scores of the invaded sites by all four groups of alien species were very similar, while the respective scores for the four groups of indigenous species differed significantly (Fig. 2a).

The PC2 extracted 19% of the variability in the dataset and was negatively correlated with the temperature variables (T_max_, T_min_). Positive correlations were found to the chemical variables: Cl, PO_4_, NO_3_, NH_4_, and O_2_, with highest positive correlation to PO_4_ and NH_4_, as well as to distance. This PC separated sites with increased temperature from sites with increased measures of water chemistry and greater distance to the next navigable waterway. PC2 singled out sites that were invaded by alien bivalves and amphipods compared with sites inhabited by the other groups of alien and indigenous species (Fig. 2b). Sites where alien bivalves and amphipods occurred were especially characterized by warmer T_min_ and T_max_.

The PC3 explained additional 14% of the variability in the dataset. Contrary to PC1 and PC2, we found no differentiation of invaded and non-invaded sites along PC3 (Table 2). Therefore, PC3 was not considered in further analysis.

### Which variables are the most important predictors for invasion risk of freshwater habitats by the different groups of alien species?

Testing the effects of all individual variables on the invasibility on a site separately, similar patterns were found for all four groups of alien species: gastropods, bivalves, amphipods, and isopods (Table 3). For all groups, we found that T_min_ was significantly increased at invaded sites in comparison to non-invaded sites. Agreement in three groups was found that T_max_ (gastropods, bivalves, amphipods) and Cl (isopods, gastropods, amphipods) were significantly increased at invaded sites, whereas O_2_ (gastropods, bivalves, amphipods) was significantly decreased at invaded sites. PO_4_ and NO_3_ were only important in two (gastropods, amphipods), respectively, in one (amphipods) taxonomic group.

**Table 3:** Results of the t-tests, comparing the eight environmental variables between invaded and non-invaded sites of the four taxonomic groups: isopods (ISO), gastropods (GAS), bivalvs (BIV), and amphipods (AMP). The environmental variables tested were O_2_, PO_4_, Cl, NO_3_, NH_4_, the mean temperature of the warmest quarter of the year (T_max_), the mean temperature of the coldest quarter of the year (T_min_), and the distance to the next navigable waterway (Distance). AS indicates significantly higher values at invaded sites, IS at sites with indigenous species only. *P*-values are given in superscript. Non-significant combinations are left blank. To adjust for multiple testing, Bonferroni-Holm corrections were applied

	Sites			
	
Variables	ISO	GAS	BIV	AMP
O_2_		IS^0.02^	IS^0.002^	IS^0.001^
Cl	AS^< 0.001^	AS^< 0.001^		AS^< 0.001^
PO_4_		AS^< 0.001^		AS^0.004^
NO_3_				AS^0.009^
NH_4_				
T_max_		AS^< 0.001^	AS^< 0.001^	AS^0.002^
T_min_	AS^0.001^	AS^< 0.001^	AS^0.004^	AS^< 0.001^
Distance		IS^< 0.001^		IS^< 0.001^

### Which variables determine the composition of species assemblage within the four different groups: amphipods, isopods, bivalves, and gastropods?

While the PCA analysis aimed at finding different pattern in the general physico-chemical degradation of invaded and non-invaded sites to access invasion risk, the CCA analysis additionally determine the relative importance of the individual variables structuring the species assemblages of alien and indigenous species from a certain taxonomic group (isopods, gastropods, bivalves, and amphipods). Consequently, CCA allows extracting species-specific distribution patterns with regard to single environmental variables.

The forward selection of environmental variables in the CCA showed that Cl was among the three most predictive environmental variables to describe the species assemblages in all four taxonomic groups (Table 4). Also the distance to the next navigable water was similarly important for all groups, were included on rank three or four in forward selection. T_min_ was among the three most important variables in amphipods, isopods, and bivalves; T_max_ in amphipods and bivalves. For the composition of gastropods, particularly low O_2_ was predictive.

**Table 4:** Results of canonical correspondence analysis (CCA) and conditional effects in forward selection of environmental variables determining the species assemblage of alien and indigenous species: amphipods, isopods, bivalves, and gastropods. Environmental variables were as follows: O_2_, PO_4_, Cl, NO_3_, NH_4_, the mean temperature of the warmest quarter of the year (T_max_), the mean temperature of the coldest quarter of the year (T_min_), and the distance to the next navigable waterway (Distance). The significance of environmental variables was tested using a forward selection and 999 Monte Carlo permutations under full model conditions. The eigenvalues of the first two canonical axis (CA) and explained variance (%) by the CAs and the order of the environmental variables including λa, *P,* and *F* are given. Significant variables are given in bold

Amphipods				Isopods				Bivalves				Gastropods			

CA	1	2		CA	1	2		CA	1	2		CA	1	2	
Eigenvalues	0.13	0.05		Eigenvalues	0.07	0.01		Eigenvalues	0.19	0.13		Eigenvalues	0.22	0.08	
Variance	5.2	7		Variance	4.6	5.5		Variance	3.2	5.5		Variance	3.1	4.2	

Variable	λa	*P*	F	Variable	λa	*P*	F	Variable	λa	*P*	F	Variable	λa	*P*	F
**Cl**	0.06	0.001	20.8	**Cl**	0.03	0.005	8.7	**T_min_**	0.1	0.001	9.1	**O_2_**	0.16	0.001	15.2
**T_max_**	0.05	0.001	13.5	**T_min_**	0.02	0.002	6.3	**T_max_**	0.09	0.001	5.0	**Cl**	0.09	0.001	8.4
**T_min_**	0.04	0.001	13.8	**Distance**	0.01	0.04	3.4	**Cl**	0.07	0.001	4.5	**Distance**	0.08	0.001	7.8
**Distance**	0.03	0.001	9.7	**NO_3_**	0.01	0.03	3.7	**Distance**	0.08	0.001	4.5	**NO_3_**	0.03	0.002	2.8
**PO_4_**	0.02	0.003	8.5	T_max_	0.01	0.14	2.0	**NO_3_**	0.07	0.001	4.6	**PO_4_**	0.02	0.03	2.4
**NO_3_**	0.01	0.001	3.9	O_2_	0	0.49	0.6	**PO_4_**	0.04	0.03	2.3	**T_min_**	0.03	0.001	2.5
**NH_4_**	0.01	0.02	3.5	NH_4_	0	0.72	0.3	**O_2_**	0.03	0.04	1.9	**T_max_**	0.02	0.01	2.0
O_2_	0.01	0.11	1.9	PO_4_	0	0.91	0.1	NH_4_	0.01	0.81	0.6	NH_4_	0.01	0.12	1.5

**Figure 3:**
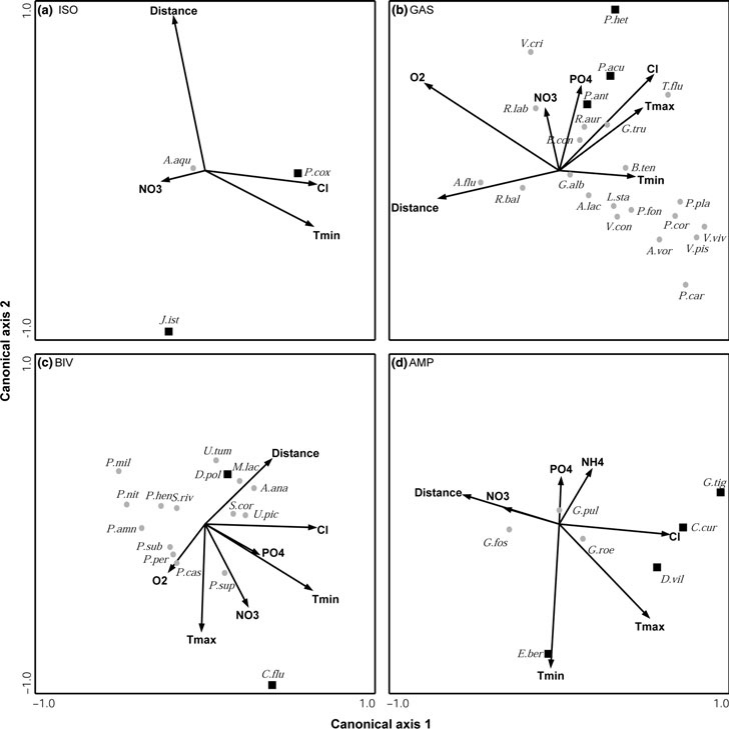
Canonical correspondence analyses (CCAs) biplots of alien and indigenous species assemblages of the four groups: (a) isopods, (b) gastropods, (c) bivalvs, (d) amphipods. For abbreviations of the species see Table 1. Black symbols indicate alien species and gray symbols indicate indigenous species. Significant environmental variables tested by forward selection and 999 Monte Carlo permutations under full model conditions are given.

In the group of isopods, Cl and T_min_ as well as the alien species *P. coxalis* were positively correlated, whereas the distance to the next navigable water, NO_3_, and the indigenous species *A. aquaticus* were negatively correlated to the first canonical axis (CA). The alien species, *J. istris* was negatively related to the second CA, which was inversely related to distance (Table 4, Fig. 3a).

The species assemblage of gastropods was significantly related to all environmental variables, except from NH_4._ Cl, PO_4_, and the temperature variables were positively related to the first CA, while O_2_, NO_3_, and the distance to the next navigable water were negatively related. All variables except from distance and T_min_ were positively correlated to the second CA. All alien gastropod species were positively related to both CAs, which means also to increased Cl, PO_4_, T_min_, and T_max_ (Table 4, Fig. 3b).

The assemblage of bivalves was significantly related to all environmental variables except from NH_4_ (Table 4). All variables except from O_2_ and T_max_ were positively related to the first CA. Furthermore, all variables except from distance were negatively related to the second CA. The alien bivalve, *C. fluminea* was also negatively related to the second CA and positively related to the first CA. In contrast, the alien bivalve, *D. polymorpha* was positively correlated to the second CA (Table 4, Fig. 3c).

The species assemblage of amphipods was significantly determined by all environmental variables except from O_2_ (Table 4, Fig. 3d). Cl, T_max_, and NH_4_ were positively related to the first CA, while distance and NO_3_ were negatively related. Further T_min_, T_max_ and Cl were negatively related to the second CA. Also the alien, gammarid *E. berilloni* was negatively related to this axis. The other alien, gammarids *G. tigrinus*, *C. curvispinum,* and *D. villosus* were positively related to the first CA (Table 4, Fig. 3d).

## Discussion

Our results showed that degraded freshwater habitats are exposed to an increased invasion risk by alien species compared with more intact habitats. The presence–absence of alien species in the four taxonomic groups containing most aliens, namely amphipods, isopods, bivalves, and gastropods was influenced by degradation and characterized by a very similar set of physico-chemical variables. In the PCA analysis, the occurrence of alien species from all four groups was related to all variables indicating environmental degradation, especially increased salinity, higher temperature, as well as lower oxygen concentrations. The reaction of indigenous members of the four respective groups to these variables, in contrast, was more variable.

This bias toward degraded habitats in the occurrence patterns of alien species reflects that alien species are a non-random set of species (Karatayev et al. 2009; Strayer 2010). Strayer (2010) lists traits that increase the chance of a species to become invasive. First, the life history strategy of different taxonomic groups is an important prerequisite to the potential to become invasive. Especially, taxa with resting stages or r-strategists with high numbers of small offspring have high invasion potential, as shown by Alonso and Castro-Diez (2008) for *P. antipodarum* and Grabowski et al. 2007) for alien gammarids. Consistently, crustaceans and mollusks are overrepresented among aquatic alien invertebrates, whereas insects that commonly have the greatest species richness in freshwater systems are underrepresented. Second, species that are readily dispersed by humans spread more easily. Thus for example, some highly persistent invaders are fishes or crustaceans that were deliberately stocked. However, most alien species were not voluntary introduced. Some species arrive as “blind passengers” by transport related to aquaculture and the ornamental trade, or are released from aquaria (Gollasch and Nehring 2006). Third, the vectors that transport alien species are highly selective. Organisms often have to tolerate harsh environmental conditions during the transport, leading to the survival of more tolerant species. The spread along shipping routes via clinging to ship hulls and carry-over in ballast water are the most powerful mechanisms for the spread of aquatic alien species. 46% of all alien species in freshwater systems in Central Europe have likely been introduced by shipping and via man-made waterways (Gollasch and Nehring 2006). The survival of a transport in ballast water tanks requires a wide tolerance to various environmental stressors. As ballast water tanks are alternately filled with fresh and salt water, organisms are exposed to fluctuations in salinity. In addition, these tanks undergo oxygen depletion and strong changes of temperature. These stressors act in the same way on the different species, which are transported in these tanks. We believe that the common tolerance to salinity, high temperature, and oxygen depletion of all alien species, disregarding their taxonomic affiliation, reflects this bottleneck in the anthropogenic introduction and establishment process of potential alien species. Non-random survival of the harsh conditions in ballast water tanks may be the most severe filter that selects particularly those species, which are well suited to thrive especially in more degraded habitats.

Sites that were invaded by alien species of all taxonomic groups except bivalves had, on average, significantly higher Cl concentrations than sites that were non-invaded. Grabowski et al. (2009) showed this pattern already for alien amphipods. Our study generalizes this previous finding showing that habitats, which are contaminated by Cl, are also more vulnerable to invasion by other aquatic taxa. Among the alien species occurring in samples that we analyzed, especially the alien amphipods *D. villosus*, *C. curvispinum,* and *G. tigrinus*, the alien isopod *P. coxalis*, all alien gastropods, as well as the alien bivalve *C. fluminea* were positively related to high Cl loads. This confirms the finding of Alonso and Castro-Diez (2008) of a selection process via anthropogenic introduction favoring euryhaline species.

The success of salt tolerant species may be further enhanced by the salt intolerance of many indigenous species. In contrast to seawater and freshwater, brackish water is characterized by the lowest number of indigenous species and is highly vulnerable to invasion (Paavola et al. 2005). Consequently, reduced numbers of indigenous species in freshwater habitats with increased salt concentration may result in increased invasion risk.

Beside increased Cl concentration, increased temperature was one of the most predictive variables for an increased invasion risk. An increase in T_min_ favored the invasibility of freshwater habitats by all four groups of alien species, as winter mortality of cold-intolerant species is reduced, as exemplified by Werner and Rothhaupt (2008) in *C. fluminea*. Also, the reproduction success of cold-intolerant species can be enhanced by warmer winter temperatures (Weitere et al. 2009). In this analysis, the occurrence of *C. fluminea* was particularly related to high T_min_. Also, the alien gammarid *E. berillioni* as well as the alien isopod *P. coxalis* were positively related to high T_min_. Warmer winter temperatures are particularly found at cooling water outlets or industrial direct dischargers. With ongoing climate change, winter temperatures are predicted to increase in many areas worldwide, which will increase the likelihood of alien species becoming established by eliminating cold temperature as an environmental filter for thermophilous species (e.g., Rahel and Olden 2008; Huang et al. 2011).

An increase in T_max_ was found to enhance the invasibility of aquatic habitats for alien species from the group of amphipods, gastropods, and bivalves. Many alien species are tolerant to higher temperatures, and often even profit from them (Wijnhoven et al. 2003; Alonso and Castro-Diez 2008). For instance, these advantages may result from modifications in species interactions. Thus, Velde et al. (2009) showed that predation success of the alien *D. villosus* on indigenous gammarids increased with temperature. Other advantages, due to increased temperature especially in the reproductive period, include increased growth rates, earlier maturation, and higher reproductive output. In our analysis, particularly the alien amphipods *G. tigrinus, C. curvispinum, D. villosus,* the alien gastropods *P. acuta, P. heterostropha, P. antipodarum,* and the alien bivalve *C. fluminea* showed a positive reaction to an increase in T_max_.

As the solubility of oxygen in water is physically linked to temperature, oxygen depletion is often, but not exclusively, linked to increased temperature. Also eutrophication and increased saprobic activity can lead to oxygen depletion. Habitats with reduced oxygen concentrations showed a higher invasion risk in all taxonomic groups except for isopods. However, especially in isopods, the reference group of indigenous species contains *A*. *aquaticus*, an extremely generalist and indicator for pollution. The dominance of amphipods in habitats with low oxygen concentrations has already been shown (MacNeil et al. 2000). Furthermore, Byers (2000b) confirmed a higher tolerance against anoxia for the alien estuarine snail *Batillaria attramentaria* compared with the indigenous *Cerithidea californica* in laboratory and field experiments. The CCA results of this study indicate that also the occurrence of the alien species, *P. acuta, P. heterostropha, P. antipodarum,* and *D. polymorpha* is related to oxygen depletion.

In contrast to all other alien species, the occurrence of *D. polymorpha* was not related to physico-chemical degradation, with the exception of low oxygen concentration. In turn, it is known that substrate structure is highly important for *D. polymorpha*. The mussel is most abundant on hard surfaces, particularly rocky surfaces, and on macrophytes. The biological interactions with a more recent invader, *C. curvispinum*, may be part of the reason for this atypical occurrence pattern of *D. polymorpha*. The presence of this alien amphipod can inhibit the occurrence of *D. polymorpha* due to the substrate modification caused by the building of its burrows (Van Der Velde et al. 1994).

Beside physico-chemical variables, the distance to the next navigable water was identified as an important predictor for invasion risk. Distant sites to navigable waters were less likely to be invaded, and also the occurrence of the single alien species was most often negatively related to this distance. With increasing distance from navigable waters, the propagule pressure of alien species decreases. The key role of the propagule pressure as a driver of invasion processes has already been acknowledged (Lockwood et al. 2009). Additionally, with increasing distance to navigable waters, also the physical habitat properties become increasingly dissimilar to the habitats available along shipping routes. Thus, species that manage to establish in shipping routes may find it increasingly difficult to find suitable physical habitats.

### Application of our results

With the results of this study, degraded sites close to navigable waters can be identified as high risk habitats for invasions of all major groups containing invasive species in Central Europe. Amelioration of physico-chemical conditions in such habitats may help to avoid such stepping stones for the further spread of alien species.

A range of studies show that once alien species are established, eradication of them is hardly feasible. Instead, alien species must be detected and removed at very early phase of their establishment, or even better, their establishment should be prevented (Strayer 2010). Therefore, understanding the variables that control the establishment and spread of alien species is essential to develop management strategies to protect habitats from invasions in order to protect native species (Lövei 1997; Dextrase and Mandrak 2006) and to prevent economic losses associated with invasions (Pimentel et al. 2005).

For this purpose, an assessment of the invasion risk of different aquatic habitats is a helpful tool. Therefore, risk assessment models are useful tools. This study demonstrates that physico-chemical habitat properties can be used to estimate invasion risk. So far, there are some studies that estimate the invasion risk of habitats for individual alien species based on species distribution models (SDMs) (e.g., Peterson 2003). The disadvantage of this approach is that current SDMs are typically based on the climate envelope of the species (air temperature and precipitation). Consequently, these models are relatively unspecific. SDMs that include water temperature, discharge, and other important physico-chemical variables, as well as data on distances to the next navigable waterway are so far lacking. We encourage to link data from hydraulic and water quality models with SDMs to get a more precise picture of invasion risk in freshwater ecosystems. Our study shows that this risk assessment does not need to be assessed for each alien species separately, but that to some degree; generalization is possible due to the similar realized niche of invasive species with respect to physico-chemical habitat degradation. Such integrated models will help to assess invasion risk more realistically.
